# The Emerging Therapeutic Potential of Nitro Fatty Acids and Other Michael Acceptor-Containing Drugs for the Treatment of Inflammation and Cancer

**DOI:** 10.3389/fphar.2020.01297

**Published:** 2020-09-03

**Authors:** Matthias Piesche, Jessica Roos, Benjamin Kühn, Jasmin Fettel, Nadine Hellmuth, Camilla Brat, Isabelle V. Maucher, Omar Awad, Carmela Matrone, Simon Gabriel Comerma Steffensen, Georg Manolikakes, Ulrike Heinicke, Kai D. Zacharowski, Dieter Steinhilber, Thorsten J. Maier

**Affiliations:** ^1^Biomedical Research Laboratories, Medicine Faculty, Catholic University of Maule, Talca, Chile; ^2^Oncology Center, Medicine Faculty, Catholic University of Maule, Talca, Chile; ^3^Department of Safety of Medicinal Products and Medical Devices, Paul-Ehrlich-Institut (Federal Institute for Vaccines and Biomedicines), Langen, Germany; ^4^Department of Anesthesiology, Intensive Care Medicine and Pain Therapy, University Hospital Frankfurt, Goethe University Frankfurt, Frankfurt, Germany; ^5^Institute of Pharmaceutical Chemistry, Goethe-University, Frankfurt am Main, Germany; ^6^Division of Pharmacology, Department of Neuroscience, School of Medicine, University of Naples Federico II, Naples, Italy; ^7^Department of Biomedicine, Medicine Faculty, Aarhus University, Aarhus, Denmark; ^8^Animal Physiology, Department of Biomedical Sciences, Veterinary Faculty, Central University of Venezuela, Maracay, Venezuela; ^9^Department of Organic Chemistry, Technical University Kaiserslautern, Kaiserslautern, Germany

**Keywords:** covalent drugs, electrophilic fatty acids, Michael acceptor, nitroalkylation, post-translational modifications

## Abstract

Nitro fatty acids (NFAs) are endogenously generated lipid mediators deriving from reactions of unsaturated electrophilic fatty acids with reactive nitrogen species. Furthermore, Mediterranean diets can be a source of NFA. These highly electrophilic fatty acids can undergo Michael addition reaction with cysteine residues, leading to post-translational modifications (PTM) of selected regulatory proteins. Such modifications are capable of changing target protein function during cell signaling or in biosynthetic pathways. NFA target proteins include the peroxisome proliferator-activated receptor *γ* (PPAR-*γ*), the pro-inflammatory and tumorigenic nuclear factor-κB (NF-κB) signaling pathway, the pro-inflammatory 5-lipoxygenases (5-LO) biosynthesis pathway as well as soluble epoxide hydrolase (sEH), which is essentially involved in the regulation of vascular tone. In several animal models of inflammation and cancer, the therapeutic efficacy of well-tolerated NFA has been demonstrated. This has already led to clinical phase II studies investigating possible therapeutic effects of NFA in subjects with pulmonary arterial hypertension. Albeit Michael acceptors feature a broad spectrum of bioactivity, they have for a rather long time been avoided as drug candidates owing to their presumed unselective reactivity and toxicity. However, targeted covalent modification of regulatory proteins by Michael acceptors became recognized as a promising approach to drug discovery with the recent FDA approvals of the cancer therapeutics, afatanib (2013), ibrutinib (2013), and osimertinib (2015). Furthermore, the Michael acceptor, neratinib, a dual inhibitor of the human epidermal growth factor receptor 2 and epidermal growth factor receptor, was recently approved by the FDA (2017) and by the EMA (2018) for the treatment of breast cancer. Finally, a number of further Michael acceptor drug candidates are currently under clinical investigation for pharmacotherapy of inflammation and cancer. In this review, we focus on the pharmacology of NFA and other Michael acceptor drugs, summarizing their potential as an emerging class of future antiphlogistics and adjuvant in tumor therapeutics.

## Introduction

Compounds possessing Michael acceptor units feature a broad spectrum of bioactivity. However, they have been largely excluded from drug discovery endeavors because of their presumed unselective reactivity and toxicity. Nevertheless, the recent FDA approval of several cancer drugs has demonstrated that covalent modifications *via* Michael addition can be a powerful tool to develop new drugs (Bauer, 2015; Ghosh et al., 2019).

Covalent modifications of proteins *via* post-translational modifications (PTMs) are a rather effective strategy to modulate protein function and activity. Such modifications include phosphorylation, acetylation, glycosylation, oxidation, and hydroxylation. Among all amino acids, cysteine plays a particularly important role in covalent modifications and is susceptible to phosphorylation, acetylation as well as oxidation. Such modifications can affect the cellular localization of the protein, its interaction with other binding partners as well as its function or activity. PTMs of proteins are of regulatory significance in almost all cell types and functional systems, including the immune system, the cardiovascular system, and the gastrointestinal system (Bürkle, 2002; Ehrentraut and Colgan, 2012; Liu et al., 2016; Fert-Bober et al., 2018).

The majority of research studies had focus on protein phosphorylation. Methylation of lysine or arginine residues, acetylation, nitrosation of thiol groups and tyrosine residues as well as alkylation of cysteines or other nucleophilic amino acids have received less attention. Alkylation of nucleophilic amino acids, including cysteine, is achieved either by reaction with alpha-halocarbonylation, aminoethylation, or by Michael addition to a molecule containing a Michael acceptor. In this review, we will focus on the Michael addition as an important reaction of approved drugs or drug candidates to induce PTMs that alter protein function.

**Figure 1:**
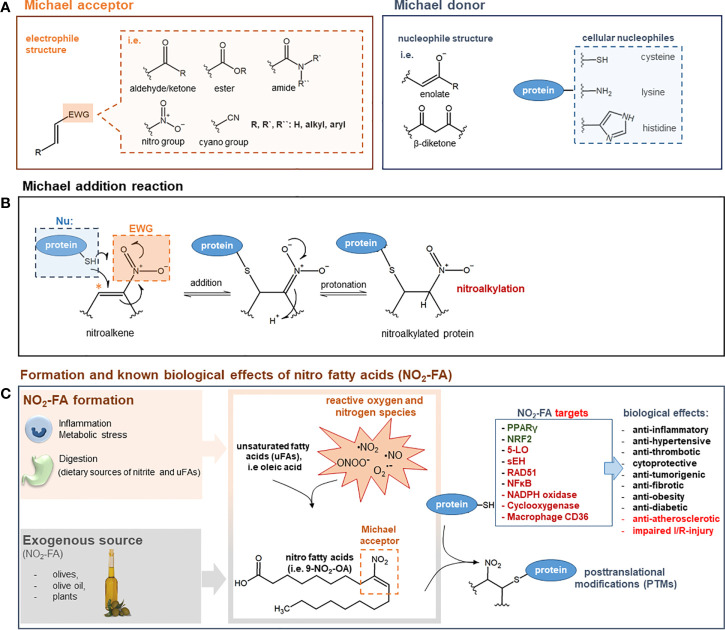
**(A)** General structure of Michael acceptors and Michael donors. Michael acceptor moiety: electron withdrawing group (EWG) adjacent to an olefin structure forming an electrophilic, electron-deficient olefin. Examples of EWGs: aldehyde, keto, ester, amide, cyano, or nitro groups. Michael donor: nucleophiles such as enolates, *β*-diketones, thiols of cysteines, imidazoles of histidines, or ϵ-amino groups of lysines. **(B)** Mechanism of Michael addition reaction. The Michael addition reaction is exemplified by the attack of a cellular nucleophile to the electrophilic *β*-carbon (*) of a nitroalkene moiety. After the addition of the thiolate anion a protonation step takes place to form a nitroalkylated protein. **(C)** Formation and known biological effects of nitro fatty acids (NO_2_-FA). NO_2_-FA can be endogenously generated during inflammation by a reaction of nitric dioxide (NO_2_) with unsaturated fatty acids. Nitric dioxide can derive from different reactive nitrogen species (i.e nitric oxide, peroxynitrite) or precursor molecules like nitrate (NO_3_^−^) and nitrite (NO_2_^−^). NO_2_-FA can also be directly supplemented as natural ingredients of olives, olive oil and plants NO_2_-FA engage in cell signaling processes *via* the post-translational modification (PTM) of nucleophilic protein targets such as 5-LO, PPAR*γ*, sEH, or NF-kB (proteins highlighted in green: activated/increased activity/expression; proteins highlighted in red: inhibited/decreased activity/expression). These PTMs induce profound changes in protein function and distribution and are therefore the leading cause for numerous biological effects. For a comprehensive overview on NFA targets and therapeutic effects see (Schopfer and Khoo, 2019). *5-LO: 5-lipoxygenase; NF-κB: nuclear factor-κB; NRF-2: nuclear factor erythroid 2-related factor 2; PPARγ: peroxisome proliferator–activated receptor γ; sEH; soluble epoxide hydrolase; I/R: ischemia/reperfusion*.

The Michael reaction is defined as a conjugate addition of a nucleophile (Michael donor) to an electron-deficient olefin, such as an *α*,*β*-unsaturated carbonyl compound (Michael acceptor) (**Figures 1A, B**). However, instead of the carbonyl group, the substituent can also be a nitro group or another strongly electron-withdrawing group. Cellular nucleophiles, *e.g.* the thiol group of cysteine, the imidazole of histidine, or the *ϵ*-amino group of the amino acid lysine have also been described to be Michael donors. Well-recognized Michael acceptors that play a major part in the resolution process of inflammation are endogenously generated anti-inflammatory electrophilic lipids called nitro fatty acids (NFAs) (Rubbo, 2013). Other electrophilic species, which are formed during inflammatory reactions, are cyclopentenone prostaglandins (*i.e.*, 15Δ-PGJ2). In this review, we will focus on NFA as representatives of lipid-derived electrophilic species, discuss other Michael acceptor-containing drugs engaged in clinical trials or already approved, and show the emerging therapeutic potential of this class of drugs.

## Nitro Fatty Acids as Naturally Occurring Mediators Containing a Michael Acceptor Moiety

Unsaturated fatty acids can be metabolized under inflammatory conditions to reactive products to act as pro- or anti-inflammatory mediators (Grimble and Tappia, 1998) (**Figure 1C**). A special group of those lipid mediators are electrophilic alkenes, like NFA. They are generated endogenously and can be detected in the plasma of human blood. Besides their endogenous generation, NFAs can also be dietary supplemented as natural ingredients of olives or native olive oil (Fazzari et al., 2014). Moreover, evidence has shown that dietary supplementation with nitrate (NO_3_^−^), nitrite (NO_2_^−^), and conjugated linoleic acid (cLA) can have an obvious effect on NFA plasma levels (Delmastro-Greenwood et al., 2015). High concentrations of reactive oxygen and nitrogen-derived species generated within inflamed tissue promote the formation of NFA. Hereafter, the nitrogen-derived species react with unsaturated fatty acids, yielding electrophilic NFA (Freeman et al., 2008). NFAs engage in cell signaling, among others, through Michael addition reactions showing distinct anti-inflammatory actions (Rubbo, 2013). The most studied NFAs are nitro-oleic acid (NO_2_-OA), nitro-linoleic acid (NO_2_-LA), nitro-conjugated linoleic acid (NO_2_-cLA), and nitro-arachidonic acid (NO2-AA). Through Michael addition, NFAs can adduct intracellular glutathione (GSH) as well as susceptible protein cysteine and histidine residues, inducing changes in protein structure, functionality, and subcellular distribution. PTM of cysteine residues by NFA has been shown to be reversible (Batthyany et al., 2006; Baker et al., 2007).

## Therapeutic Effects of NFA

The protective and beneficial effects of NFA could be demonstrated in a number of *in vivo* animal disease models. Thus, therapeutic effects were proposed for the following diseases:

### Classical Inflammatory Diseases

Inflammation is part of the body’s immune defense responses. However, inflammatory processes require complex regulation to warrant a local and temporal restriction of inflammation and avoid chronification potentially triggering some types of cancers, rheumatoid arthritis, periodontitis, asthma, and Crohn’s disease. NFAs have been shown to modulate directly the activity of a number of pro-inflammatory enzymes or factors involved in the acute phase of inflammation, such as nuclear factor-*κ*B (NF-*κ*B, see section *NF-κB*), 5-Lipoxygenase (5-LO, see section *5-Lipoxygenase*), and Prostaglandin endoperoxide H synthases 2 (COX-2, see section *Prostaglandin endoperoxide H synthases 1 and 2*). However, NFA mediated effects not only might contribute to symptom relief but also to active enhanced resolution of inflammation. Resolution of inflammation includes abrogation of immune cell recruitment at sites of inflammation, removal of activated immune cells, and suppression of production of pro-inflammatory mediators. NFAs affect resolution by triggering activation of the resolution factor transcriptional factor peroxisome proliferator-activated receptor *γ* [PPAR*γ*, see section *PPARγ* and for overview on the factors role in resolution see (Croasdell et al., 2015)]. The direct covalent binding of NFA to functionally important amino acid residues of inflammatory target proteins might facilitate strong and sustained pharmacological impacts. Furthermore, reactions with amino acids which are poorly conserved among closely related proteins and embedding of the target amino acids into specific clefts can increase selectivity of binding of covalent drugs to an exclusive set of proteins (Singh et al., 2011) which might potentially also apply to NFA. This might allow targeting a unique set of regulatory key proteins in inflammation. The known target proteins mediating the anti-inflammatory effects of NFA are listed in section *Nucleophilic Targets Susceptible to Michael Addition by NFA to Explain Their Therapeutic Effects*.

Several *in vivo* models have shown a therapeutic effect of NFA in preclinical models of inflammation, *e.g.*, pretreating mice with NFA in a model of LPS-induced inflammation resulted in a reduced severity of multiorgan dysfunction compared to LPS alone. Expression of inflammatory mediators in the NFA-treated group was also reduced compared to the LPS group (Wang et al., 2010). In a model of inflammatory bowel disease, the addition of NFA resulted in attenuated colonic inflammation and improved the clinical symptoms of this disease. The activation of PPAR*γ* played an important role in this protection (Borniquel et al., 2010). However, the route of NFA administration seems to be important as demonstrated by Mather et al. They showed in a model of allergic contact dermatitis (ACD) that the administration of NFA subcutanously induced an immunosuppressive responses, including an increased activity of regulatory T cells (Mathers et al., 2017). In contrast, a topical administration of NFA in the same mouse model exacerbated the inflammatory response, including the infiltration of neutrophils, inflammatory monocytes, and *γδ* T cells (Mathers et al., 2018).

### Cardiovascular Diseases

By 2030, it is expected that cardiovascular disease (CVD) will account for 25 million deaths worldwide. Even in underdeveloped countries, CVD surpasses infectious diseases, indicating a high medical need for new treatment options (Okwuosa et al., 2016). CVDs including hypertension, coronary heart disease, and atherosclerosis are potentially associated with an elevated generation of reactive oxygen species (ROS) and nitric oxide (NO) and compromised endogenous antioxidant defenses (Mann et al., 2009), suggesting that NFA could be important players in CVD. Indeed, a number of publications indicate that NFAs possess protective effects against CVD. Exemplified, NFA-induced endothelium-independent vasorelaxation, which possibly involves release of NO (Freeman et al., 2008). Furthermore, in animal models of atherosclerosis, NFAs have been shown to reduce infarct size, decrease neutrophil infiltration into the infarct zone to prevent myocyte apoptosis (Rudolph et al., 2010b), reduce lipid accumulation, and promote plaque stability (Rudolph et al., 2010a). Finally, antihypertensive effects of NFAs have been reported, *e.g.*, nitro-oleic acid inhibits angiotensin II-induced hypertension (Zhang et al., 2010; Charles et al., 2014; Klinke et al., 2014).

### Cancer

Globally, cancer is the second leading cause of death. Inflammatory processes are crucially involved at all stages of tumor development, starting from tumor initiation, promotion, malignant transformation, tumor invasion, and tumor metastasis. Furthermore, some targets of NFA are well-recognized players in tumorigenesis, and oxidative stress modulates these different stages of inflammation-induced carcinogenesis. Thus, a role of NFA in tumorigenesis has been proposed. Recently, it has been demonstrated that NFAs suppress the growth of breast cancer by diminishing cancer cell viability along with tumor cell migration and invasion (Woodcock et al., 2018). Furthermore, NFAs have been shown to enhance the cytotoxic activity of DNA-damaging agents on growth of triple-negative breast cells and might therefore function as adjuvants in therapy of such types of cancers (Asan et al., 2019). Finally, NFAs suppress tumor growth by causing mitochondrial dysfunction and activation of the intrinsic pathway of apoptosis in colorectal cancer cells. Inhibition of the pro-inflammatory proteins, NF-*κ*B and 5-lipoxygenase, which are involved in tumorigenesis, is considered a possible mode of action for NFAs, explaining their chemopreventive effects (Kühn et al., 2018).

### Fibrosis

Fibrosis is characterized as the overgrowth and hardening of a connective tissue in response to an injury or damage. The precise pathophysiological mechanism of generation of fibrosis is rather complex and still unknown; however, there seems to be a connection between fibrotic events and chronic inflammation (Wynn, 2008). In 2014, Reddy et al. reported that nitro fatty acids abolished pulmonary fibrosis and reduced disease severity in a mouse model with a possible role of NFA-mediated activation of PPAR (Reddy et al., 2014). Recently, NFAs have been demonstrated to protect against steatosis and fibrosis during development of non-alcoholic fatty liver disease in mice fibrosis (Rom et al., 2019). Moreover, NFAs have been reported suppressing angiotensin II-mediated fibrotic remodeling and atrial fibrillation with mechanisms that still need further investigation (Rudolph et al., 2016). NFAs might therefore be a novel lipid-based therapeutic strategy against different types of fibrotic processes with molecular mechanisms that need to be addressed in future studies.

## Nucleophilic Targets Susceptible to Michael Addition by NFA to Explain Their Therapeutic Effects

NFAs are potent electrophiles that alkylate susceptible thiols of multiple transcriptional regulatory proteins, affecting downstream gene expression and modulating metabolic as well as inflammatory signaling pathways (Trostchansky et al., 2013). In a study done by Khoo and Li et al., the effects of different NFA derivatives on the NF-kB and Nrf2 signaling pathways were investigated to better understand NFA structure–function relationships. This study demonstrated that NFA derivatives having varying carbon chain lengths and different positions of the nitroalkene group and show different potencies in affecting the above-mentioned signaling pathways (Khoo et al., 2018). Moreover, Gorczynski and Smitherman et al. demonstrated that the potency of NFA to activate PPAR-*γ* may vary according to the position of the NO2 group, where the position of nitration plays an important role in optimal PPAR-*γ* activation (Gorczynski et al., 2009). A number of protein targets of NFA have already been identified, which might explain some of the therapeutic effects of NFA (**Figure 1C**).

### PPAR *γ*

The transcriptional factor PPARγ is a nuclear receptor regulating lipid homeostasis, inflammatory signaling, and adipocyte differentiation. PPAR*γ* activation in myeloid cells suppresses the expression of pro-inflammatory mediators, like interferon-*γ* (IFN*γ*) and nitric oxide synthase (iNOS or NOS2) (Tontonoz and Spiegelman, 2008). PPAR*γ* has also been associated with neutrophil apoptosis along with clearance and resolution of inflammation (Konopleva et al., 2004). NFAs are partial agonists of PPAR*γ*. For that reason, they can restore insulin sensitivity *in vivo*. Furthermore, unlike Rosiglitazone they cause no weight gain while reducing the insulin and glucose levels in Lep^ob/ob^ mice. Therefore, this feature is considered as an advantage that highlights its beneficial actions and potentially reduces the adverse effects associated with full PPARγ agonists (Schopfer et al., 2010; Lamas Bervejillo et al., 2020). They are also weaker agonists of PPAR-*α* and *β*/*δ* (Baker et al., 2005; Schopfer et al., 2005). It has been shown that PPAR*γ* agonists appear to have direct neuroprotective actions in several different animal models, like Alzheimer’s disease (AD), stroke, multiple sclerosis (MS), Parkinson’s disease (PD), and amyotrophic lateral sclerosis (Sundararajan et al., 2006). Therefore, activation of PPAR*γ* could explain some of the anti-inflammatory and possible neuroprotective actions of NFAs. NFAs used as drugs might therefore be useful for the therapy of these diseases.

### NF-*κ*B

Nuclear factor-*κ*B (NF-*κ*B) plays a significant part during inflammatory responses and is involved in the initiation, development, metastasis, and resistance to the treatment of cancer. In unstimulated cells, NF-*κ*B dimers are sequestered in the cytoplasm by the inhibitor of *κ*B proteins, I-*κ*B (canonical/classical NF-*κ*B pathway). Upon activation, I-kB releases NF-*κ*B, allowing it to translocate into the nucleus where it activates the transcription of pro-inflammatory cytokines and other inflammatory mediators. NF-*κ*B is comprised of two subunits, *i.e.* p50 and p65 (Mitchell et al., 2016). NFAs can specifically nitroalkylate the p65 subunit of NF-*κ*B and, to a lower extent, the p50 subunit. Alkylation inhibits the translocation and DNA-binding affinities of NF-*κ*B and, in consequence, inhibits its pro-inflammatory activities (Cui et al., 2006; (Khoo et al., 2018). This causes the repression of NF-*κ*B dependent target gene expression and cytokine production such as tumor necrosis factor *α* (TNF*α*), interleukin-6 (IL-6), monocyte chemoattractant protein 1 (MCP-1) and the vascular cell adhesion molecule 1 (VCAM-1) that plays an important role in monocyte rolling and adhesion which is essential for the inflammatory process (Cui et al., 2006; Villacorta et al., 2018). In line with these findings, NFAs were reported to suppress the growth of aggressive breast cancer cells by inhibiting NF-*κ*B transcriptional activity, thereby suppressing downstream NF-*κ*B target gene expression (Woodcock et al., 2018). Furthermore, NFAs are able to interfere with the initial toll-like receptor-4 (TLR4) signaling upstream of the NF-*κ*B cascade by disrupting the recruitment of the receptor into lipid rafts and assembly of the adaptor protein TRAF6/IKKb/IkBa complex in vascular cells. However, the exact mechanism still remains to be defined (Villacorta et al., 2013). Inhibition of NF-*κ*B could therefore play a certain role in NFA-induced anti-tumorigenic effects as well as in some of the NFA-induced anti-inflammatory effects.

### Nrf2-Keap1

Nuclear factor erythroid 2-related factor 2 (Nrf2) is a transcription factor that regulates the expression of genes encoding for proteins that counteract oxidative stress triggered during cell and tissue injury and inflammation. Under basal conditions, the transcription factor Nrf2 is suppressed by cytosolic Keap1 (Kelch-like ECH-associated protein 1), which promotes rapid ubiquitination and proteasomal degradation of Nrf2 (Kobayashi and Yamamoto, 2005). NFA activate Nrf2-dependent antioxidant gene expression by nitroalkylation of the thiol residues of critical cysteines, such as Cys273 and 288, in the Nrf2 regulatory protein, Keap1, thus facilitating the translocation of Nrf2 into the nucleus (Kansanen et al., 2011). The expression of Nrf2-dependent genes, including heme oxygenase-1 (HO-1), glutathione peroxidase (GPx), glutathione reductase, glutathione S-transferase, or superoxide dismutase then promotes cell protection by attenuating the inflammatory response (Zhu et al., 2008; Dreger et al., 2010). Glutathione (GSH) itself is a tripeptide synthesized from glutamate, cysteine, and glycine. GSH is catalyzed by two cytosolic enzymes, which are *γ*-glutamylcysteine synthetase and GSH synthetase. GSH metabolism plays a crucial role in the defense against oxidative stress, nutrient metabolism, and regulation of cellular events essential for whole-body homeostasis (Wu et al., 2004). Regarding HO-1, nitrolinoleic acid was able to induce its activity in a cell culture model of pulmonary epithelial cells as well as in the lungs of rats (Iles et al., 2009). The therapeutic effects of activating Nrf2 through NFA could be useful for treatment of oxidative stress- and Nrf2-dependent diseases, such as cancer and several types of inflammatory and neurodegenerative diseases.

### 5-Lipoxygenase

Lipoxygenases (LO) catalyze the generation of reactive lipid mediators derived from arachidonic acid, such as leukotrienes and 5-HETEs. These oxidized products support inflammatory processes by acting as chemotactic and chemokinetic agents as well as bronchioconstrictive factors (Haeggström and Funk, 2011). Our own studies have demonstrated that nitro-oleic acid is a potent inhibitor of 5-LO *in vitro* and *in vivo*. This effect is because of a nitroalkylation of catalytically relevant cysteine residues, C416 and C418, resulting in a loss of enzyme activity (Hörnig et al., 2012; Awwad et al., 2014). Blocking 5-LO was a major mechanism responsible for the suppression of lipopolysaccharide-induced pulmonary inflammation in mice dosed with NFA (Awwad et al., 2014). The 5-LO-inhibitory potency of NFA could be beneficial for the treatment of pulmonary diseases, such as bronchial asthma, but also for cardiovascular diseases and cancer with the well-documented pathophysiological role of this enzyme (Steinhilber et al., 2010). In this sense, treatment of pulmonary hypertension could be a promising target.

### Soluble Epoxide Hydrolase

Another enzyme that possesses a reactive cysteine, which is essential for catalytic functioning, is soluble epoxide hydrolase (sEH). sEH catalyzes the hydration of epoxides which is crucial for the regulation of blood pressure by the modulation of epoxyeicosatrienoic acid (EET) levels and their influence on blood vessel relaxing tonus through the named endothelial hyperpolarization mechanism. The conserved cysteine residue, C521, which resides proximal to the catalytic center of sEH can be alkylated by electrophilic lipids, leading ultimately to the inhibition of the enzyme (Charles et al., 2011). In a C521S sEH redox-dead knock-in mouse model, it was shown that treatment with NFA protects mice from hypertension only with sEH wildtype C521. Mice with an sEH C521S mutation did not benefit from NFA treatment, suggesting an underlying Michael reaction of NFA with this cysteine (Charles et al., 2014).

**Figure 1** summarizes the nucleophilic targets of NFA and their potential role in disease.

### Microsomal Prostaglandin E2 Synthase-1

Microsomal prostaglandin E synthase 1 (mPGES1) is a terminal enzyme of the cyclooxygenase pathway which catalyzes the last step of the synthesis of the pro-inflammatory mediator prostaglandin E_2_ (PGE_2_). The isoprostane 15-deoxy-Δ12,14-prostaglandin J_2_ (15d-PGJ2) is a naturally occurring degradation product of prostaglandin D_2_ which is another bioactive product of the cyclooxygenase pathway. Notably, 15d-PGJ_2_ is not a member of the class of nitro fatty acids as the Michael acceptor moiety consist of a cyclopentenone motif lacking a nitro group. Interestingly, Prage and Jakobsson et al. could demonstrate that 15d-PGJ_2_ can inhibit mPGES1 by covalent modification of residue C59 and by noncovalent inhibition through binding at the substrate (PGH_2_) binding site which can potentially explain some anti-inflammatory actions of 15d-PGJ_2_ (Prage et al., 2012).

### Prostaglandin Endoperoxide H Synthases 1 and 2

Prostaglandin endoperoxide H synthase is an important enzyme that catalyzes the conversion of arachidonic acid (AA) to prostaglandin G2 (PGG2) and its subsequent reduction to prostaglandin H2 (PGH2), which is expressed during inflammation. PGHS exists in two isoforms, PGHS-1 and -2, which are found in mammalian tissues. Trostchansky et al. demonstrated that nitration of the carbon chain of AA yields novel nitroarachidonic acid isomers with new biological properties and causes the diversion of arachidonic acid from its normal metabolizing pathways. Nitroarachidonic acid inhibited peroxidase activity in PGHS-1 and -2 (COX-1 and 2) as well as oxygenase activity in PGHS-1. In addition, both isoforms, PGHS-1 and -2, were unable to use nitroarachidonic as a substrate for oxygenase or peroxidase activity. These effects suggest their potential pharmacological relevance during inflammation (Trostchansky et al., 2011).

### CD36

The membrane protein CD36 is the latest protein, identified as direct NFA target in macrophages (Vazquez et al., 2020). The CD36 protein is expressed on the surface of various cell types including immune cells and mediates long-chain fatty acid and cholesterol ester uptake among other functions. Binding of NFA to CD36 reduced mLDL (modified low density lipoprotein) uptake and both cholesterol and cholesteryl ester accumulation in macrophages potentially providing an explanation for the NFA-mediated athero-protective effects animal models.

## Further Approved or Clinically Developed Therapeutic Drugs Containing Michael Acceptors

**Figure 2:**
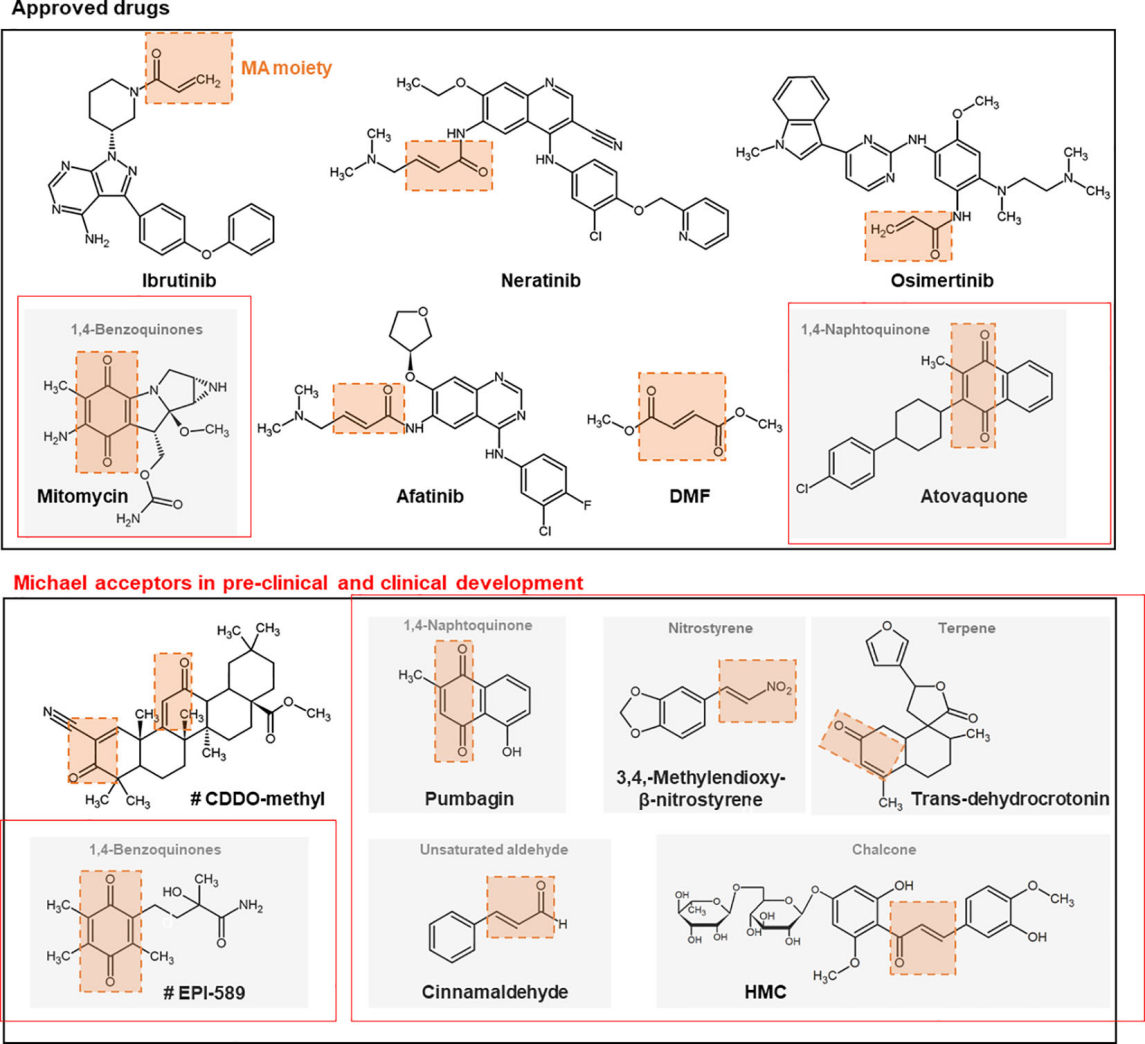
Chemical structures + of different approved and (pre-) clinically developed therapeutic drugs containing Michael acceptors. The Michael acceptor moiety is highlighted in orange. Compounds marked with # are currently studied in clinical trials. *CDDO, 2-cyano-3,12-dioxo-oleana-1,9(11)-dien-28-oic acid; DMF, dimethyl fumarate; MA, Michael acceptor; HMC, hesperidin methyl chalcone*.

Examples of further synthetic and naturally occurring Michael acceptors that target noncatalytic cysteine thiols are described subsequently. Structures of drugs or structural scaffolds with the Michael acceptor moiety highlighted in red are shown in **Figure 2**.

### Approved Drugs

#### Ibrutinib

Ibrutinib is an inhibitor of Bruton’s tyrosine kinase (BTK) with well-recognized antineoplastic activity. For BTK inhibition, the drug uses a Michael acceptor moiety for irreversible binding to the target cysteine (Herman et al., 2011). Such inhibition induces a modest cancer cell apoptosis, abolishes proliferation, and prevents both B-cell activation and B-cell-mediated signaling. Ibrutinib was FDA-approved in 2013 for mantle cell lymphoma and later for chronic lymphocytic leukemia (CLL, 2014) and B-cell lymphoma-like Waldenström macroglobulinemia (Castillo et al., 2016). In 2017, ibrutinib was approved as a second-line chronic graft *versus* host disease (cGVHD) (Miklos et al., 2017). In addition, ibrutinib has also been shown to have potential effects against autoimmune arthritis (Akinleye et al., 2013). Some patients have had reported relapse during ibrutinib therapy, which was based on an acquired resistance to this drug, based in most cases of cytogenetic abnormalities (Byrd et al., 2020). Functional studies indicate that the C481S mutation in *BTK* is the reason for resistance to ibrutinib by preventing irreversible drug binding (Woyach et al., 2014).

#### Neratinib

Neratinib is an irreversible tyrosine-kinase inhibitor of epidermal growth factor receptor (EGFR)/human epidermal growth factor receptor 1 (HER1), HER2, and HER4. Neratinib comprises a quinolone core that reacts through a Michael addition with the same reactive substituents as afatinib (see the following) but with an affinity and pharmacological potency that is lower (Feldinger and Kong, 2015). The inhibition of tyrosine kinases lead to a G1-S phase arrest, which results in inhibition of tumor cell proliferation (Rabindran et al., 2004). It has been shown that neratinib is less potent in inhibiting proliferation of EGFR-positive cells compared to HER2-positive cells (Canonici et al., 2013). Furthermore, it has been demonstrated that neratinib can reverse membrane-bound ATP transporter-mediated multidrug resistance (Zhao et al., 2012). Neratinib was approved in 2017 as adjuvant treatment for patients with early-stage HER2-overexpressed/amplified breast cancer. Neratinib is further being evaluated in clinical trials for advanced/metastatic breast cancer and solid tumors, including HER2-mutated tumors (Feldinger and Kong, 2015).

#### Osimertinib

Osimertinib is a third-generation EGFR tyrosine kinase inhibitor that was marketed in 2017 to treat advanced or metastatic non-small-cell lung cancer (NSCLC) carrying a specific mutation. The drug targets cancer cells that contain the T790M mutation in the gene coding for EGFR but spares cancer cells with wildtype EGFR (Lategahn et al., 2019). However, within a time period of approximately 1 year, cancer cells can become resistant through various mechanisms, such as amplification of cMet and HER2. Nevertheless, the main mechanism of resistance to osimertinib is the mutation of the non-catalytic cysteine (C797S), representing the target amino acid of the drug for the Michael reaction (Patel et al., 2017). Therapeutic strategies to overcome osimertinib resistance are described elsewhere (Tang and Lu, 2018).

#### Afatinib

Afatinib is a protein kinase inhibitor that was approved in 2013 for the treatment of NSCLC. The chemical drug contains an electrophilic group capable of a Michael addition reaction to conserved cysteine residues inside the catalytic domains of EGFR, HER2, and HER4. This reaction inhibits irreversible enzymatic activity (Solca et al., 2012). Afatinib has also been investigated for breast cancer because of its additional activity against HER2 (Minkovsky and Berezov, 2008). However, a clinical phase II trial has indicated there is no benefit from afatinib alone or when combined with the microtubule assembly inhibitor vinorelbine (Jim Yeung, 2005) compared with treatment of the investigator’s choice in women suffering from HER2-positive breast cancer with progressive brain metastases during or after therapy with trastuzumab, lapatinib, or both (Cortés et al., 2015).

#### Dimethyl Fumarate (DMF)

DMF is the ester of the unsaturated dicarboxylic fumaric acid. A number of *in vitro* and *in vivo* studies have shown a potent anti-inflammatory effect of DMF in a variety of diseases, *e.g.*, MS (Kappos et al., 2008), psoriasis (Nieboer et al., 1989; Reich et al., 2009), and asthma (Seidel et al., 2009). Currently, oral DMF is approved for MS (2013) and psoriasis (2017). The results of two large phase III trials testing DMF in remitting MS led to its rapid regulatory approval, first by the US Food and Drug Administration (FDA) in 2013 and then by the European Medicines Agency (EMA) in spring 2014 (Fox et al., 2012; Gold et al., 2015). In 2017, EMA approved an oral formulation of DMF for the treatment of adult patients with moderate-to-severe chronic plaque psoriasis (Mrowietz et al., 2018).

Similar to NFA, DMF can alkylate Keap1, which leads to a stabilization and translocation of Nrf2 (Seidel et al., 2009; Linker and Haghikia, 2016). Induction of Nrf2-mediated gene expression is considered the major mode of action responsible for suppression of neurodegenerative processes of MS. Additionally, Gillard et al. showed that DMF treatment led to significant inhibition of the nuclear translocation of p65 (canonical/classical NF-*κ*B) and p52 (non-canonical NF-*κ*B) signaling (Gillard et al., 2015) with a therapeutic relevance that requires further evaluation.

### Michael Acceptors in Pre-Clinical and Clinical Development

#### Bardoxolone Methyl (Also Known as CDDO-Methyl Ester or RT-402)

CDDO-methyl ester (CDDO-Me), a semi-synthetic triterpenoid derived from oleanolic acid, is a promising chemotherapeutic and anti-inflammatory agent in clinical development (Couch et al., 2005; Celentano et al., 2019; Rossing et al., 2019; Tian et al., 2019). The structure of CDDO is comprised of two *α*, *β*-unsaturated carbonyl moieties, which are accessible for nucleophilic addition. An essential factor for potency is not the triterpenoid skeleton but the cyanoenone group whose absence greatly reduces the activity of CDDO-Me. This Michael acceptor structure can generate reversible adducts with cysteine residues in target proteins like Keap1 and IkB kinase, leading consequently to an activation of the NRF2/Keap1 pathway and inhibition of NF-*κ*B signaling (Wang et al., 2014). Interestingly, the selective binding of CDDO-Me to cysteine residues of different proteins seems to be both context-dependent and dose-dependent. It has been shown that low concentrations of CDDO-Me protect cells against oxidative stress whereas higher concentrations are known to induce apoptosis (Wang et al., 2014). CDDO-Me and the related analog inhibit inflammatory responses and tumor growth *in vivo* and have also been considered for use in patients (Place et al., 2003). In particular, CDDO-Me underwent phase III clinical trials for chronic kidney disease (CKD) as well as phase I/II clinical trials for malignant diseases (Wang et al., 2014). However, it was discontinued for CKD owing to an increased risk of heart failure (de Zeeuw et al., 2013). Nevertheless, it is still being tested in clinical trials for treatment of obesity in adult men (NCT04018339, phase I), pulmonary hypertension (NCT03068130, phase III), chronic or diabetic kidney diseases (NCT03749447, phase III, and NCT03550443, phase III, respectively), Alport Syndrome, (NCT03019185, phase II/III) and autosomal dominant polycystic kidney disease (NCT03918447, phase III). Omaveloxolone (*N*-(2-Cyano-3,12-dioxo-28-noroleana-1,9(11)-dien-17-yl)-2,2-difluoropropanamide; CDDO- DFPA) is being further tested for the treatment of patients with Friedreich’s Ataxia (NCT02255435, phase II).

#### Polyphenols

Polyphenols are major constituents of many herbal remedies exhibiting anti-inflammatory activities (González et al., 2011). They are characterized by the presence of multiple phenol structural units. Different types of polyphenols also contain a Michael acceptor unit within their structure. In addition, the oxidation of the parent polyphenol can lead to the formation of a reactive olefin, such as in the oxidation of a hydroquinone to a quinone. Over the last decade, there has been abundant attention to the possible health benefits of dietary plant polyphenols as antioxidants (Pandey and Rizvi, 2009). It has been reported that a number of polyphenolic extracts suppress tumor cell proliferation and reduce pro-inflammatory processes by inhibiting 5-LO (Leifert and Abeywardena, 2008), NF-*κ*B, and mitogen-activated protein kinase signaling (Santangelo et al., 2007). The inactivation of NF-*κ*B by polyphenols is thought to be mediated by their interaction with cysteine residues in either I*κ*B kinase or the DNA-binding domain of NF-*κ*B, particularly the Cy38 of the p65 subunit (Wang and Dubois, 2010). However, the beneficial effects of polyphenols have been mainly demonstrated by *in vitro* studies. Several factors, like low bioavailability, poor solubility, and high metabolism of some of the polyphenols, may account for the poor and difficult clinical translations of these compounds (Christensen, 2018). Chalcones, an example of polyphenols, are discussed subsequently.

#### Chalcones

Chalcones demonstrate a broad and versatile spectrum of pharmacological activities, including immunomodulation, anti-inflammatory, anticancer, antiviral, and antibiotic properties (Lee et al., 2015). Recently, a stable topical formulation has been tested containing the chalcone derivative hesperidin methyl chalcone (HMC) protecting the skin of mice towards UVB-induced oxidative stress and inflammation (Martinez et al., 2017). Other Chalcones have recently been identified as 5-LO inhibitors and urenyl chalcone derivatives exert a dual inhibition of cyclooxygenase-2 (COX-2)/5-LO activities (Lee et al., 2015). Further studies could show that chalcone derivatives inhibit secretory phospholipase A_2_, COX enzymes, lipoxygenases, pro-inflammatory cytokine synthesis, neutrophil chemotaxis, immune cell phagocytosis, and production of ROS (Bukhari et al., 2014a; Bukhari et al., 2014b; Lee et al., 2015). Recently, a novel chalcone derivate (chalcone-*O*-alkylamine derivate) has been documented, demonstrating that it might be a multifunctional anti-AD agent (Bai et al., 2019). Besides all the promising potential of chalcone derivates, there are no approved drugs available to date.

#### Nitrostyrenes

3,4-Methylenedioxy-*β*-nitrostyrene has been identified as a NOD-, LRR-, and pyrin domain-containing protein 3 (NLRP3) inflammasome inhibitor with a Michael addition as the proposed mode of action. The activation of the NLRP inflammasome triggered caspase-1 activation and the release of the cytokine interleukin-1*β*, a pro-inflammatory mediator, which is involved in both acute as well as chronic inflammatory responses. Thus, NLRP3 has been implicated in the pathogenesis of several human diseases, such as gout, silicosis, type I/II diabetes, general endothelial dysfunction, erectile dysfunction, atherosclerosis, and AD (Baldwin et al., 2016; Pereira et al., 2019; Fais et al., 2019). Furthermore, the suppression of the inflammasome may efficiently reduce damaging processes, such as K+ efflux, lysosomal membrane destabilization, ROS generation, and ubiquitin/deubiquitination post-translational modifications (Baldwin et al., 2016). Therefore, the NLRP3 inflammasome is an attractive therapeutic target (Baldwin et al., 2016). Moreover, nitroalkene analog of *α*-tocopherol have been designed for the prevention and treatment of inflammation related diseases (Rodriguez-Duarte et al., 2018).

#### Quinones

1,4-benzoquinones are recognized for their anti-inflammatory, antioxidative, and anticancer activities (Schaible et al., 2014). A popular representative of this group is the active ingredient thymoquinone, isolated from *Nigella sativa* (Woo et al., 2012). Anti-inflammatory effects have been found to be associated with suppression of leukotriene formation (Werz, 2007) and 1,4-benzoquinone AA-861 is a well-recognized 5-LO inhibitor (Yoshimoto et al., 1982). EPI-589, a (R)-troloxamide quinone, is currently in clinical trials for PD. The estimated completion date was December 2019, but no results have been published as of yet (NCT02462603). Mitomycin, a benzoquinone, is used in the clinic for non-invasive or minimally invasive bladder cancers, and in combination with 5-fluorouracil (5-FU) as well as radiation during treatment of stage I-III anal cancer (Milla et al., 2014). Notably, mitomycin also contains a pharmacologically active aziridine group that leads to alkylations of target proteins.

#### Naphthoquinone

Naphthoquinone forms the structural basis of a number of natural compounds, most pre-eminently the K vitamins. Naphthoquinones are known for their antibiotic, antiviral, antifungal, antiphlogistic, and antipyretic properties (Hernández-Pérez et al., 1995; Kobayashi et al., 2011). The naphthoquinone plumbagin is a naturally compound in the medicinal herb *Plumbago zeylanica*. This herb has been safely used for centuries in Indian Ayurvedic and Oriental medicine for treating various ailments, including bacterial infections and allergic processes (Powolny and Singh, 2008). Furthermore, plumbagin has already been described to suppress NF-κB activation (Sandur et al., 2006). Plumbagin can also reduce the viability of human prostate cancer cells by triggering apoptosis. Adding N-acetylcysteine (NAC) significantly attenuated this effect (Powolny and Singh, 2008) indicating that the reaction of plumbagin with cellular proteins containing thiol groups might play an important role in the pharmacological activity of plumbagin. The naphthoquinone atovaquone is used to treat or prevent, *e.g.*, pneumocystis pneumonia (PCP) (only mild cases), toxoplasmosis, and malaria where it is one of the two component drugs along with malarone (National Institute of Diabetes and Digestive and Kidney Diseases, 2012).

#### Unsaturated Carboxylic Acids and Aldehydes

Unsaturated carboxylic acids and aldehydes are a structurally rather heterogeneous group. Examples are either NFA or cinnamaldehyde. NFA and their targets are described in detail above. Cinnamaldehyde is the main constituent of cinnamon. Cinnamaldehyde is a pleitropic bioactive compound that attracted lots of interest for its anticancer, anti-inflammatory, antidiabetic, and antifungal properties. It has also been reported to be beneficial against neurological diseases, *e.g.*, PD and AD (Rao and Gan, 2014). Cinnamaldehyde contains an *α,β*-unsaturated aldehyde and can act as a Michael acceptor. It is a potent activator of the transient receptor potential cation channel, subfamily A, member 1 (TRPA1) (Sandur et al., 2006), a Ca^2+^ channel that plays an important role in inflammatory and neuropathic pain, as well as the pathogenesis of AD (Lee et al., 2016). More detailed information about cinnamaldehyde and its potential as therapeutic agent is reviewed in Chen et al. (2017).

#### Terpenes

Some terpenes, which is the largest group of phytochemicals, contain a Michael acceptor unit (Butturini et al., 2011), *e.g.*, trans-dehydrocrotonin and crotonin. Both compounds originate from croton plants from the Amazonian region and have been associated with anti-inflammatory, anti-atherogenic, and anti-ulcerogenic properties (Hiruma-Lima et al., 2002). Furthermore, other diseases that affect the cardiovascular system, such as diabetes, have been shown to have positive effects from aqueous extracts of the stem barks of Croton cuneatus Klotz, which significantly reduced blood glucose levels in diabetic rats (Torrico et al., 2007).

A vast number of many other terpenes (Vasas and Hohmann, 2014) and phenolic (Kris-Etherton et al., 2002) compounds have been shown to possess protective effects regarding the cardiovascular system, including relaxation in conductance vessels, antithrombotic properties, lowering low-density lipoproteins (main cholesterol transporter for atheroma formation), or reduction of coronary heart disease and cardiovascular risk factors, as well as reversal of endothelial dysfunction.

These findings could indicate that the Michael acceptor moiety and reactivity of the drugs with thiols of target proteins are relevant to the therapeutic effects triggered by terpenes.

#### Therapeutic Effects of the Endogenous Michael Donor, GSH, and GSH Inhibitors

GSH is an abundant natural tripeptide found within almost all cells at concentrations of 0.5 to 10 mM (Lushchak, 2012). Oxidative stress can lead to chronic inflammation, which in turn could mediate most chronic diseases (Reuter et al., 2010). GSH is vital for protecting tissues against the degenerative effects of oxidative damage through the conjugation of chemically reactive electrophilic molecules from endogenous or exogenous agents and thus preventing unwanted reactions with important cell constituents (Reed, 1986; Lu, 1999). The Michael addition is one of the mechanisms how GSH protects nucleic acids and proteins from these agents. Endogenous agents are described extensively by Wang and Ballatori (Wang and Ballatori, 1998). One example is the electrophilic eicosanoids, which contain *α,β*-unsaturated ketones and are biosynthesized during the oxidative metabolism of arachidonic acid. GSH adducts have been observed with molecules derived from lipoxygenases (Wang and Ballatori, 1998) and with electrophilic fatty acids (Batthyany et al., 2006).

GSH plays also an integral role in the clearance of drugs. The aforementioned drug, afatinib, undergoes extensive conjugation with GSH both in buffer and cytosol fractions deriving from liver and kidney tissues, whereas ibrutinib has exhibited much lower degree of GSH-dependent conjugation (Shibata and Chiba, 2015). The importance of GSH in drug clearance can be seen when patients accidentally take an overdose, *e.g.*, acetaminophen, a medication used to treat pain and fever, which is generally safe when used in the recommended dosage. However, when taken in overdose, it can cause a potentially fatal, hepatic centrilobular necrosis (James et al., 2003), which accounts for almost one-half of all patients with acute liver failure in the United States and Great Britain. At nontoxic doses, the metabolite of acetaminophen is efficiently detoxified by GSH, forming an acetaminophen-glutathione conjugate *via* Michael addition (Jollow et al., 1974). However, at toxic doses, the metabolite depleted hepatic GSH by as much as 80–90% (Mitchell et al., 1973; Jollow et al., 1974). Repletion of GSH using an antidote like N-acetylcysteine was able to prevent toxicity (Dargan and Jones, 2003).

Another important function of GSH is in the detoxification of small toxic molecules, thereby rendering them into less toxic derivatives. This activity accounts for one type of drug resistance, a key element in the failure of chemotherapy treatment. GSH can be combined with anticancer drugs to yield less toxic GSH conjugates with a higher water-solubility. The GSH conjugates of chemotherapeutics can penetrate out of the cells by the glutathione S-conjugate export (GS-X) pump or multidrug resistance-associated protein (MRP). Levels of GSH, glutathione-related enzymes, and the GS-X pump or MRP have been demonstrated to be elevated or overexpressed in a number of drug-resistant tumor cells (Zhang et al., 1998). A number of inhibitors to block or downregulate GSH to increase tumor responsiveness to chemotherapy are under investigation in several clinical trials (Trachootham et al., 2009; Singh et al., 2012). The most advanced drug is currently undergoing Phase III trials where the GSH inhibitor APR-246 and azacitidine or azacitidine alone is being compared in patients with TP53-mutated MDS (NCT03745716).

## Therapeutic Options for Michael Acceptors for Neurodegenerative/Neuroinflammatory Diseases

A link between Michael acceptors and neurodegenerative diseases has largely been established by a variety of sources. As Michael acceptors are present in the manufacturing, agricultural, and polymer industries, human exposure to these compounds is pervasive. Indeed, acrolein and methylvinyl ketone (MVK) are environmental pollutants while acrylamide (ACR) and methyl acrylate are dietary contaminants (reviewed in (Morgan et al., 2000; Friedman, 2003). Michael acceptors, because of their metastable and reactive properties, attack synaptic proteins and form complexes that accumulate at the nerve terminals. Consistently, elevated levels of acrolein and 4-hydroxy-nonenal (HNE) have been found in the degenerating neurons of the substantia nigra of PD patients (Yoritaka et al., 1996), where it has been hypothesized that they promote α-synuclein aggregation. Similarly, *α,β*-unsaturated aldehydes are generated endogenously as a break-down product of lipid peroxidation of *ω*-6 polyunsaturated fatty acids and have been thought to be responsible for synaptotoxicity and nerve terminal dysfunction in PD (Kehrer and Biswal, 2000; Friedman, 2003; LoPachin et al., 2008; Lee and Park, 2013; Fecchio et al., 2018). In AD, patients experienced increases in both *α* and *β* secretase levels, which has been linked to the presence of various lipid peroxidation products, including MDA, F2-isoprostanes, and HNE. Overall, elevated levels of Michael acceptor derivatives have also been detected in amyloid plaques (Markesbery and Carney, 1999). Evidence of lipid peroxidation was detected in Huntington’s brain tissues, where, in particular, HNE was found to colocalize with Huntington inclusions (Lee et al., 2011).

In addition, ACR, acrolein, HNE, and other unsaturated carbonyl derivatives inhibit NO signaling at the nerve terminal, triggering neuroinflammatory processes, a common feature shared by most neurodegenerative diseases (Csala et al., 2015).

Interestingly, Michael acceptors have recently gained interest for their potential therapeutic properties with respect to neurodegenerative disease.

In this regard, recent studies have underlined the cytoprotective effects against oxidative stress that the synthetic triterpenoid (TP) derivatives of CDDO exert within either *in vivo* or *in vitro* animal model of neuronal deficits (Dinkova-Kostova et al., 2005; Tran et al., 2008; Dumont et al., 2009; Castellano et al., 2019). In particular, Dumont et al. found that three-month administration of CDDO-MA improved cognitive performance and reduced A*β* protein levels, which is the main component of senile plaques as well as plaque deposition in AD mouse models, by reducing inflammation, enhancing phagocytosis of the Aβ protein and plaques, and decreasing oxidative stress (Dumont et al., 2009).

Of note, additional studies pointed on the use of Bruton tyrosine kinase inhibitors in the treatment of AD as well as in MS (Montalban et al., 2019; Keaney et al., 2019). In addition, cysteine-targeting compounds such as ICE-like cysteine protease inhibitors (caspase I inhibitors) have been recently suggested as anti-apoptotic and anti-inflammatory agents to treat AD and PD patients, in which progressive neuronal death seems to be associated with caspase overactivation (LoPachin et al., 2008). The rationale of the use of Michael acceptors comes from the idea of developing compounds selectively targeting cysteine residues on caspase, thereby taking possible advantage of the low occurrence of cysteine residues in the human proteome (2.3%) and thus potentially lessening off-target effects. However, as these compounds have multiple biological activities, the possibility to use them in the treatment of neurodegenerative diseases is far from clear, thus raising scepticism in the scientific arena (Parvez et al., 2018; Poganik and Aye, 2020).

## Further Diseases With a Possible Therapeutic Efficacy of Michael Acceptors

Beside the therapeutic effects described herein, Michael acceptors might play a potential role in a number of other diseases.

Recently, antidepressive effects of certain Michael acceptors have been reported. DHIPC, a 2′-hydroxy-4′,6′-diisoprenyloxychalcone derivate, exhibits antidepressant effects by increasing serotonin, noradrenaline, and 5 hydroxyindoleacetic acid levels in the hippocampus, hypothalamus, and brain cortex of DHIPC-treated mice (Zhao et al., 2018).

The plant-derived Michael acceptor curcumin possesses multiple modes of action. The drug is able to suppress liver fibrosis by modulation of a specific miRNA mediating the epigenetic regulation of liver fibrosis (Zheng et al., 2014). Furthermore, it is an effective treatment for idiopathic pulmonary fibrosis owing to inhibition of collagen secretion, fibroblast proliferation, and differentiation (Smith et al., 2010).

The Michael acceptor epalrestat is a reversible aldose reductase inhibitor preventing the conversion of glucose to sorbitol within the polyol pathway. In Japan, it is an approved drug for treatment of subjective and objective symptoms of diabetic neuropathy, the most common long-term complication in patients suffering from diabetes mellitus (Ramirez and Borja, 2008).

Antiparasitic properties are also benefits of Michael acceptor-containing drugs. K777 is an irreversible inhibitor of cruzan, a cysteine protease of *Trypanosoma cruzi*, which causes Chagas disease. Hybrid compounds comprising an electrophilic warhead and Michael acceptor-containing structure motif (*e.g.*, vinyl sulfone, pyrimidine nitrile group) are effective anti-malaria agents by targeting the parasitic food vacuole of *P. falciparum* within the low nanomolar range (Vale, 2016). Various plant-derived Michael acceptors also exhibit activity against leishmaniasis, such as the cyclopentenedione derivate, DCPC, isolated from *Piper carniconnectivum* roots or dihydrochalcones isolated from *P. elongate* (Paes-Gonçalves et al., 2012).

Furthermore, a number of drugs containing Michael acceptors exhibit anti-viral properties. Naturally occurring compounds like 15d-PGJ_2_, celastrol, curcumin, and rosmarinic acid have well-documented anti-retroviral activity by targeting the Cys-rich domain of HIV-1 Tat, leading to inhibition of Tat-dependent transcription (Narayan et al., 2011). Rupintrivir (AG7088) was a promising Michael acceptor drug candidate that inhibits human rhino virus by targeting rhinoviral protease 3CP. Unfortunately, its development was discontinued during clinical phase II/III based on lack of efficiency in natural infection studies (Vale, 2016).

## Discussion: Future Directions and Concluding Remarks

Over the past decades, the development of inhibitors covalently binding enzymes or other target proteins *via* Michael reaction was deprioritized by the pharmaceutical industry. This was mainly because of safety concerns about indiscriminate and unselective reactivity of the covalent-modification drugs with potentially off-target proteins, thereby causing unpredictable toxicity. However, recently, several efficient and safe covalently binding inhibitors of protein kinases have been successfully approved for cancer treatment, changing the perspective on this class of drugs. Binding of noncatalytic cysteine residues with acrylamides and other *α*, *β*-unsaturated carbonyl compounds is currently the preferred strategy used for the development of Michael acceptor-containing drugs. There is rising agreement that covalent binding of target proteins using Michael acceptor moieties can improve pharmacodynamic properties, such as efficacy, potency, selectivity, and duration of pharmacological effects. However, to avoid toxicity, the scaffold encompassing the electrophilic warhead needs rather careful, prolonged, and sophisticated drug design, including computational and molecular modelling methods applied. As such, designed covalent inhibitors might possess significant advantages over non-covalent inhibitors such that covalent warheads can target unique residues of selected target proteins with a higher pharmacodynamic efficacy and less susceptibility to the phenomenon of drug resistance. Along with biomedical drugs, covalently binding inhibitors on the basis of Michael acceptor moieties might therefore play a pivotal role in the drug market of the 21^st^ century.

## Author Contributions

MP, JR, and TM contributed to the design and conception of the review. MP wrote the first draft of the manuscript. BK, JF, NH, CB, CM, SC, GM, IM, OA, UH, KZ, DS, and TM wrote sections of the manuscript. All authors contributed to the article and approved the submitted version.

## Funding

This work was funded by the Else Kröner-Fresenius-Foundation (EKFS) as well as the Graduate School TRIP (Translational Research Innovation-Pharma), the German Research Foundation (DFG project MA-5825/1-2), the DFG Sonderforschungsbereich SFB-1039, and the Aarhus University Research Foundation (AUFF). TM was recipient of a Heisenberg fellowship from the German Research Foundation (DFG-MA-5825/2-1).

## Conflict of Interest

The authors declare that the research was conducted in the absence of any commercial or financial relationships that could be construed as a potential conflict of interest.
